# The Early Notification of Births Act

**Published:** 1907-11-09

**Authors:** 


### The Hospital

A Journal of

The Medical Sciences and Hospital Administration.

New Series. No. 33, Vol. II. [No. 1105, Vol. XLIII.] SATURDAY, NOVEMBER 9, 1907.

### The Early Notification of Births Act.

When this Act was yet under discussion in Parliament, and the chance existed of modifying its provisions, we ventured, with some insistence, to urge that, however admirable its object, the machinery by which this was to be attained was certain to place the medical profession in a most objectionable position. Admitting that possibly some general social value was to be obtained by the notification of every birth within thirty-six hours of its occurrence, we objected to the proposal to place the responsibility for such notification upon the shoulders of the medical profession. Broadly our objection was that it was contrary to the public interest to put the medical practitioner in a position in which he would appear to announce publicly what he had learned in his capacity as the patient's private medical adviser. Even though supported by the sanction of legal enactment, such conduct, we argued, must inevitably tend to disturb the confidential relations which it is so important should exist between practitioner and patient, and must be most objectionable as involving a breach of professional confidence and the invasion of the highest professional traditions. Another objection, and a perfectly reasonable one, as it seems to us, was to the proposal to place the duty of notification on the profession without any corresponding recognition in the form of a fee. But, fee or no fee, we held, and expressed the view, that the Bill was thoroughly vicious in so far as it placed the responsibility of notification on the private and confidential medical adviser of the patient. Unfortunately, when the position was argued in Parliament, the professional position was defined as concerned solely with the question of the fee; and those who spoke on behalf of the profession practically said that were this granted their objection to the Bill would disappear. Even in more decided fashion, this was also the attitude assumed by the so-called representative meeting of the British Medical Association, and was proclaimed on their behalf as the voice of the profession. In these circumstances it is not a matter for wonder that Parliament made short work of these sordid protests, and passed the clauses in question without essential modification.

The Early Notification of Births Act has now become part of the law of the land, but instead of being universal and compulsory, as at first proposed, its adoption in any particular district depends on the decision of the local authority. There is therefore still opportunity for the medical profession to prevent the operation of the unhappy proposal to make practitioners public purveyors of their patients' secrets. This opportunity, we are glad to say, is being recognised, and its recognition affords an interesting commentary on the claim of those who, in "representative" meeting or in the lobbies of the House of Commons, presumed to define the attitude of the medical profession. Thus, at Manchester, a deputation of medical practitioners has waited on the sanitary authorities and has urged the non-adoption of the Act on the identical grounds which we, at the outset, presented to our readers, and which were practically ignored by the self-proclaimed "representatives" of the profession. Dr. Helme, in introducing the deputation, protested against the suggestion that the non-provision of a fee was the basis of the profession's objection to the Act. The claim he advanced was based upon the contention that to compel the medical attendant "to divulge the secrets of the sick-room" was both to force a breach of professional confidence and to put in peril individual and public interests. Exactly the same point is enforced in a letter issued to the local press by the Medical Guild of Manchester. In this, as is right and proper, the public concern in the maintenance of the rule of professional secrecy is admirably presented, and, indeed, the question is one in which patients not less than members of the medical profession are interested. "To convert the medical attendant into a State detective," says the Guild, "is not good policy, and his acquiescence in such a *rôle* . . . would not meet with his patients' approval. For the sake of the public and for our own conscience sake such an infringement of professional secrecy must be resisted." That is admirably phrased, and we hope to see the lead taken by the profession in Manchester generally adopted throughout the country.

There can be little or no doubt that when the position is fairly presented to laymen they will see how unfair, both to practitioner and patient, the compulsory provisions of the Act as they now exist must in operation prove to be. Some evidence of this is afforded by what has already taken place at Gateshead. There the local division of the British Medical Association has discussed the question with the local Health Authority, and as a result the Town Council has decided against the adoption of the Act. At the same time the local members of the profession have expressed their readiness to encourage voluntary notification of births, in cases where this seems advisable. No one questions that in certain circumstances this may be a highly advisable proceeding, but whether it should be enforced on all is another matter. In any event the position of the medical profession is clear. Their essential objection to the Act is that it demands the betrayal of professional confidences, and these, in the public interest, ought to be held as sacred.

